# Concealed pregnancy as an act of care? A qualitative analysis of motivations for concealing and non-disclosure of early pregnancy in The Gambia

**DOI:** 10.1186/s12884-023-05710-6

**Published:** 2023-05-24

**Authors:** Sabine Parrish, Senthil K. Vasan, Fredrik Karpe, Polly Hardy-Johnson, Ousman Jarjou, Mustapha Bittaye, Andrew M. Prentice, Stanley Ulijaszek, Modou Jobe

**Affiliations:** 1 School of Anthropology and Museum Ethnography, University of Oxford, 51/53 Banbury Road, Oxford, OX2 6PE UK; 2 Oxford Centre for Diabetes, Endocrinology and Metabolism, University of Oxford, Oxford, UK; 3 NIHR Oxford Biomedical Research Centre, OUH Trust, Oxford, UK; 4 University of Southampton, Southampton, UK; 5 MRC Unit The Gambia at LSHTM, Banjul, The Gambia; 6 Department of Obstetrics and Gynaecology, Edward Francis Small Teaching Hospital, Banjul, The Gambia; 7 University of The Gambia, Banjul, The Gambia

**Keywords:** Diabetes, Child health, Maternal health, Witchcraft

## Abstract

**Background:**

A barrier to achieving first trimester antenatal care (ANC) attendance in many countries has been the widespread cultural practice of not discussing pregnancies in the early stages. Motivations for concealing pregnancy bear further study, as the interventions necessary to encourage early ANC attendance may be more complicated than targeting infrastructural barriers to ANC attendance such as transportation, time, and cost.

**Methods:**

Five focus groups with a total of 30 married, pregnant women were conducted to assess the feasibility of conducting a randomised controlled trial to evaluate the effectiveness of early initiation of physical activity and/or yoghurt consumption in reducing Gestational Diabetes Mellitus in pregnant women in The Gambia. Focus group transcripts were coded through a thematic analysis approach, assessing themes as they arose in relation to failure to attend early ANC.

**Results:**

Two reasons for the concealment of pregnancies in the first trimester or ahead of a pregnancy’s obvious visibility to others were given by focus group participants. These were ‘pregnancy outside of marriage’ and ‘evil spirits and miscarriage.’ Concealment on both grounds was motivated through specific worries and fears. In the case of a pregnancy outside of marriage, this was worry over social stigma and shame. Evil spirits were widely considered to be a cause of early miscarriage, and as such, women may choose to conceal their pregnancies in the early stages as a form of protection.

**Conclusion:**

Women’s lived experiences of evil spirits have been under-explored in qualitative health research as they relate specifically to women’s access to early antenatal care. Better understanding of how such sprits are experienced and why some women perceive themselves as vulnerable to related spiritual attacks may help healthcare workers or community health workers to identify in a timely manner the women most likely to fear such situations and spirits and subsequently conceal their pregnancies.

## Background

Early antenatal care (ANC) attendance (within the first trimester) is associated with fewer complications during pregnancy and delivery, reduced maternal and infant mortality, and better life course outcomes for children [1, 2]. The World Health Organization recommends that, for best outcomes, ANC be initiated before the 12^th^ week of a pregnancy, and that pregnant women should have eight contacts with ANC healthcare workers throughout the duration of their pregnancy [3]. The consequences of late ANC attendance is a global concern not restricted only to lower- and middle-income countries [4, 5], although there have been many targeted efforts to increase ANC attendance in sub-Saharan Africa (SSA) and Asia in particular, due to the historically high rates of maternal and child mortality [6].

The Gambia is one nation in SSA which has made recent substantial progress in improving maternal and childhood survival rates, now sitting in the mid-range of maternal mortality rates for the region. Active interventions in the nation are aimed at further encouraging ANC uptake and increasing total contacts between women and healthcare workers across the duration of a pregnancy [7–9]. One difficulty in encouraging early ANC uptake in The Gambia has been the widespread cultural practice of not disclosing pregnancies in the early stages, which extends to a reluctance to disclose to family, co-wives, friends, and healthcare workers [10, 11].

Previous analysis of Gambian women’s feeling toward ANC showed that women generally prefer to book for early ANC and understood benefits of doing so, although there are frequent practical barriers to doing so [12]. These barriers have been identified by Gambian women as including: limited support from husbands [13], financial and time barriers to travel [14], caretaking and domestic responsibilities [11], and concerns about side-effects from medication [15]. Our participants echoed these concerns, but some women referred additionally to instances when women might not attend ANC because, although aware of their own pregnancy, they did not wish to disclose their pregnancies to others, including healthcare workers. Although recent work on concealed pregnancy in The Gambia has encouraged health workers and qualitative researchers to improve the detection of pregnancy in women who may outright state they are not pregnant for any reason [16], instances and motivations for concealed pregnancy themselves bear further analysis and study, as the relevant interventions necessary to encourage early ANC attendance for concealing mothers may be more complicated than those targeting practical, infrastructural barriers to ANC attendance (e.g., reimbursement of bus fare for women travelling to clinics).

## Methods

### Design

The data analysed in this study emerged from focus groups which were collected in support of a Randomised Control Trial (RCT) evaluating the effectiveness of early initiation of physical activity and/or yoghurt consumption in reducing Gestational Diabetes Mellitus (GDM) in pregnant women [17]. The focus group discussions (FGDs) analysed herein were conducted to understand Gambian women’s interest in booking early for ANC, lifestyle changes, and undergoing interventions and procedures required for participating in the wider study, along with barriers to recruitment and interventions. We believe that the outcomes from these FGDs will guide recruitment and retention for the wider RCT which is planned in the future.

Focus groups were chosen to obtain insights into mothers’ perceptions and experiences of booking for ANC and potential participation in the RCT. Focus groups were preferred over in-depth interviews or nominal group technique because a range of divergent views and experiences were sought, rather than individual views or a consensus opinion on a single issue [18].

### Study setting

The focus groups were conducted at the Kanifing General Hospital in The Gambia. Kanifing General Hospital is a tertiary referral facility, located in the urban Kanifing Municipality, the most densely populated settlement in The Gambia [19]. Outside of the national capital, Banjul, Kanifing Municipality has the highest proportion of births occurring in medical facilities [20]. The majority of the population in The Gambia are Muslim, although in practice this is often blended with local spiritual beliefs [9, 21].

### Participant sampling and recruitment

The FGDs were covered under the wider RCT’s feasibility study ethical clearance. All methods were performed in accordance with the Declaration of Helsinki’s guidelines. The study was approved by The Gambia Government/MRCG joint ethics committee (SCC 1620).

We used convenience sampling to recruit participants for the focus group discussions. Pregnant mothers attending an antenatal clinic at Kanifing General Hospital were approached for their participation. Study staff (social scientist and research clinician) approached women and explained the objectives and details of the study before obtaining informed consent. An informed consent form was administered by the research clinician or field worker facilitating the FGD sessions. The consent form was written in English and was verbally translated as required. The participants were allowed as much time as wished to consider the information, and the opportunity to question the personnel administrating the ICF. Written informed consent was obtained by means of participant dated signature and dated signature of the person who administered the consent form. In cases of illiterate participants, her left thumb impression was obtained. Those women who consented when approached by researchers at the antenatal clinic were then invited to a room within the antenatal facility for the focus group discussions.

### Study population

Five FGDs were conducted in February 2019 with 30 married pregnant women aged 16–37 years old who reported between one and six pregnancies (including their current pregnancy) (see Table 1). The only information recorded about women participating in FGDs were their ages and numbers of pregnancies (including current), and therefore the sample may not be representative of the local population as a whole, though all three of the most commonly spoken languages in The Gambia were represented.

**Table 1:** Participant characteristics

FGD	Participant ages (years)	Number of participants in FGD	Number of pregnancies per participant (including current)
1	19–37	6	1–5
2	23–29	6	1–5
3	16–32	6	1–4
4	19–35	6	1–5
5	19–30	6	1–6

### Data collection

A pre-written FGD guide was used to facilitate the discussions. The guide (Table 2) contained questions about willingness to participate in the proposed RCT from 12–14 weeks of gestational age, routine antenatal care, proposed interventions, adherence to study protocol, retention, factors that may restrict adherence to study protocol and acceptability of the yoghurt as well as the procedures around physical exercise.

**Table 2:** Focus group discussion guide

Willingness to book for ANC in the first trimester
Willingness to participate in a diet (yoghurt consumption) and physical activity intervention trial
Willingness to undergo study-related procedures (laboratory test, ultrasound scan, using electronic arm band, walking during pregnancy and measurements of baby)
Barriers that limit participation of pregnant women to yoghurt consumption and physical activity
Barriers to protocol adherence

FGDs were facilitated by two Gambian researchers: an experienced female social scientist, who has successfully collected data on many similar studies with pregnant Gambian women, assisted by a male research clinician. Four were conducted in both the Mandinka and Wolof languages (as participants commonly spoke both languages), with one FGD conducted exclusively in Fula—these are the most commonly spoken languages in The Gambia. Sessions were recorded with participants’ permission, and the audio recordings were later transcribed by the trained field worker who led the interviews, and translated into English. The transcribed texts were independently reviewed (together with the recordings) by two study staff who were fluent in the local languages of the recordings and English. The discussions lasted an average of two hours.

### Patient and public involvement

Patients or the public were not involved in the design, conduct, or reporting plans of this research but the preliminary outcome of the findings were considered for the final draft of protocol of the RCT. A summary of the main trial results will be disseminated to the general public and study participants after the completion of the study.

### Data analysis

The analysis in this paper focuses specifically on women’s answers relating to FGD theme number one, ‘willingness to book for ANC in first trimester’, and the ways in which beliefs can be barriers to intervention and protocol adherence. Transcripts were uploaded into NVivo (v12) and, using Braun and Clarke’s thematic analysis approach [22], the first author coded the transcripts, deductively generating themes as they arose in relation to failure to attend early ANC, with women’s concealment of pregnancy emerging as a strong theme which had not previously been analysed in relation to this data set. This initial framework was presented to and verified by authors two through nine, with intercoding for verification performed by author eight, and showed that in addition to the infrastructural and relational barriers associated with late ANC attendance previously identified, there were reasons which would cause women to actively conceal their pregnancy from others, with the result that they would not attend ANC for fear of disclosure.

## Results

Two reasons for the concealment of pregnancies in the first trimester or ahead of a pregnancy’s obvious visibility to others, were given by FGD participants. These were ‘pregnancy outside of marriage’ and ‘evil spirits and miscarriage’. Concealment on both grounds was motivated through specific worries and fears. In the case of a pregnancy outside of marriage, this was worry over social stigma and shame. Evil spirits are understood as a cause of miscarriage in early pregnancy, and as such, women may choose to conceal or not disclose their pregnancy as a protection mechanism.

### Pregnancy outside of marriage

FGD participants reported that women who found themselves pregnant outside of marriage were understood to be less likely to attend early ANC for fear of running into acquaintances at the clinic and the pregnancy being revealed. This theme was spoken about in generalised terms with no specific examples presented.

Respondent: *Some women get pregnant out of wedlock. These women feel shy to show up in the clinic and be in the same place with other pregnant women. They also avoid to be seen by their family members while attending ANC. They don’t want others to say to their family members “I have seen Mrs X at the antenatal clinic. Is she pregnant?” —*FGD5

FGD participants did not elaborate on what the social consequences of such a pregnancy out of marriage might be; these results will be considered in the Discussion in relation to extant literature on the social consequences of pregnancy outside marriage. Further, all FGD participants were married, and none indicated that they had concealed a pregnancy for this reason.

R*: Am sorry to say this, there are pregnant women who feel ashamed of attending ANC because they got pregnant out of wedlock. They don’t want to be seen by people. Excuse me for my language. This could be one of the main reasons why they book late, but if you are married you are good to go. —*FGD4

### Evil spirits and miscarriage

The more prevalent discourse related to the concealment of pregnancy was linked to local cultural beliefs around the causes of miscarriage. Many of the women interviewed held the view that making one’s pregnancy known to others at an early stage places one at greater risk of miscarriage as pregnancy makes one vulnerable to attacks by evil spirits. The term ‘*kunto jinno*’ (‘evil spirit’; derived from the Arabic ‘*jinn*’, for ‘spirit’) was that which was commonly used in Mandinka. ‘*Kunto fengo*’ (‘evil thing’) was also employed by Mandinka speakers, as there is a belief among some individuals that one should avoid using the word ‘*jinno*’ so as to not draw a spirit’s attention to oneself. *‘Raab*’ was the term employed by Wolof speakers. Each of these terms refers to a type of spirit who is neither divine nor an ancestor, but which occupies the same landscapes as and has complex relationships with humans.

Fear of early disclosure of pregnancy leading to miscarriage resulted in some women keeping their pregnancies secret and not attending ANC.

R: *Such things do occur in our communities*. *There are women if they conceive, they have miscarriages once people notice that they are pregnant. Once people notice that some women are pregnant, they will have a miscarriage.* —FGD5

Divergent from the general, unspecified way in which pregnancy out of marriage was spoken about, these spiritually induced miscarriages were known to have happened to specific individuals:

R: *There is a lady I know who is afflicted with such a problem. It is these types of evil spirits that always abort her pregnancies. Whenever she is pregnant, she becomes hopeful but only to lose it when her pregnancy is about three months. Whenever she goes to the toilet, the evil spirits flush out the whole conceptus into the toilet. […] it is the evil spirits she is possessed by who are the culprits. She tries very hard to hide her pregnancies but loses them once they reach three months. You know this is painful. […] My sister-in-law is possessed by such spirits. She loses her pregnancies once they are three months old.* —FGD4

Indeed, one FGD participant, in response to a general comment from another participant, reported suffering from miscarriages attributable to these spirits and early disclosure of pregnancy:

R1: *Yes, they used to say that some women have spiritual forces and once people detect that they are pregnant during their first month, they can easily have a miscarriage or it will hunt them during labour. This is why others hide their pregnancy until their delivery is due.*

R2*: Yes, it happens because even I am a victim; I had a miscarriage in my first pregnancy when I was three months. This is why many women don’t like people knowing that they are pregnant early. […] It is a natural thing in some women. Once people detect that they are pregnant, they easily experience a miscarriage.* —FGD2

However, some women were aware of the belief that spirits were the cause of miscarriages but did not ascribe to it, although they were aware that it influenced the actions and choices of other women. In the following exchange, the second respondent notes how a miscarriage may occur because a woman has not attended early ANC, but then perceived as being caused by spirits.

R1: *Yes, this [that women may lose their pregnancies on account of spirits] is true for some women once people start talking about their pregnancy while the pregnancy is in its early stage. […] In most cases they will wait until the pregnancy is obvious before they book for ANC.*

R2*: I don’t think this should prevent pregnant women from booking for ANC. It is better to book early when your pregnancy is about three months old, otherwise you might have a miscarriage and it will be attributed to evil spirits. –FGD3*

## Discussion

This qualitative analysis of FGD data considered the reasons for which women in urban Gambia might choose to conceal a pregnancy. It identified ‘pregnancy outside of marriage’ and ‘evil spirits and miscarriage’ as two distinct reasons a woman may wish to conceal a pregnancy, including from healthcare workers by not attending early ANC.

Women were understood to conceal those pregnancies which occurred outside of marriage for as long as possible. Previous research in The Gambia has shown that not wanting to be the subject of gossip and avoiding the stigma of unwed motherhood drive this type of concealment among women of various ethnic groups [10, 23]. Women who become pregnant while not married will conceal until a marriage can be arranged, an abortion accessed, or until the pregnancy is visually obvious and no longer able to be hidden [24]. While FGD participants referenced gossip, they did not directly discuss the question of shame. Pregnancy outside of marriage is understood as in violation of appropriate codes of conduct, and such pregnancies may be seen as both shameful for the woman in question as well as her wider family, jeopardising not only the marriage prospects of the pregnant woman but also any sisters she may have [25].

Regarding the question of miscarriage attributable to evil spirits, participant familiarity with both the phenomenon and individuals who have suffered such miscarriages indicates that far from an isolated or fringe cultural belief, the presence of spirits continues to inform lived pregnancy experiences for many women in The Gambia as it does other aspects of daily living. Indeed, as anthropologist Susannah Chapman has described for The Gambia, ‘it is not uncommon for *jinn* to intercede, assist, or even interfere with the affairs of humans’, be they pregnant women or otherwise [26]. Our participants invoked the presence of *jinn* in relation to other discussion topics, suggesting that the proposed physical activity interventions of the RCT must take into account the times of day when *jinn* are most active and therefore when it is not appropriate for an individual to be outdoors; for these urban women *jinns* were not outdated folk beliefs, but rather, had an active presence in their experiences of daily life. That traditional beliefs and practices continue to exist together with awareness and utilisation of biomedical services—even in highly urbanized areas of The Gambia [11]—suggest that more robust understandings of the cultural contexts in which pregnancy concealment occurs would aid in interventions which are both effective and locally-sensitive. 

If public disclosure of a pregnancy is understood to be extremely dangerous to the life of the foetus on account of harm from spirits, active concealment and reluctance to speak about a pregnancy may be read not as negligence born of ignorance of biomedical treatments and interventions, but rather, a considered act of care practiced by the woman for the benefit of her motherhood. Study participants were aware that early ANC attendance would have certain beneficial health outcomes. Yet some women continued to conceal pregnancies for the threat of evil spirits, revealing that this fear of spiritually-induced miscarriage can outweigh the recognized benefits of attending ANC early. In other words, though traditional beliefs and biomedical knowledge around causality may exist together in The Gambia, that does not mean they are weighted equally in all situations.

While several studies have identified concerns about evil spirits as a driver for women to conceal pregnancies in Gambia and elsewhere across SSA [27], the context of evil spirits have been under-explored in in-depth qualitative terms as they relate specifically to ANC access. Better understanding of how such spirits are held to operate, drawn from anthropological literature and ethnographic studies, may help healthcare practitioners identify the women most likely to fear such situations and who accordingly choose to conceal their pregnancies. An example of concealment as a deliberate and considered act of care in a similar context is given by anthropologist Rachel Chapman, who has explored the intersection of evil spirits and pregnancy in Mozambique [28, 29]. She shows how pregnant women are vulnerable to the threat of spirits—caused most frequently by jealous individuals, such as co-wives or other relatives—precisely because pregnancy is a period of heightened social risk for women [30, 31]. The Mozambican case parallels what we find in The Gambia, with women fearful of being the target of evil spirits and refusing early ANC seeing this as a ‘preventive and protective health activity’ which mitigates the more immediate, acute threats of pregnancy loss [32]. With motherhood a key social role for adult women in The Gambia, recurrent miscarriage and failure to bring pregnancies to term can destabilise women’s standings within families, leaving them socially, psychologically and economically vulnerable; protecting against pregnancy loss is an essential activity to ensure a woman’s long-term stability [33, 34]. There is ethnographic evidence of supernatural afflictions causing neonatal death in rural Gambia [35] and authors note that pregnant women are particularly vulnerable to spiritual attacks causing a range of illnesses [36] yet the exact reasons behind why pregnant women’s vulnerability remain absent in the literature and call for further study.

Both the fear of social stigma associated with unwed motherhood and fear of miscarriage caused by evil spirits can lead to late ANC attendance on account of non-disclosure of pregnancy in the early months. The invocation of such terms and beliefs as ‘spirits’ may appear to some biomedical practitioners as irrational or without basis. However, we can consider evil spirits as a motivation for concealment to exist, like social stigma related to being an unmarried mother, as a reflection of the multiple considerations, both medically and culturally informed, mothers must entertain in the name of caring for and protecting their unborn children. Further, there have been some noted links between gossip about a pregnancy (as was discussed by participants in relation to unmarried mothers) and miscarriage: gossip can alert evil spirits to the pregnancy in the first place, thereby making the mother a target [10]; this bears further scrutiny, as it suggests the motivations for concealment we have described may, in some instances, be linked rather than discrete concerns.

We call attention to the ways in which Gambian women’s concealment of pregnancy shares characteristics with other pregnancy (non) disclosure strategies already substantially accounted for in academic and clinical literature in relation to the Global North, and which all share the characteristic of being a way to manage uncertainties and fears [37]. While the rare and dramatic cases of pregnancy hidden until birth draw greatest attention [38], most concealment in Global North parallels that of The Gambia, in that it is typically confined to the first trimester: while women generally share pregnancy status with both their partners and healthcare practitioners, cultural norms nevertheless dictate that wider family and society should not normally be informed of the pregnancy until the second trimester. Whatever their motivation, women not disclosing pregnancies in the early months evade drawing attention to themselves and invent alternate explanations of symptoms [39], as is described of Gambian women [10]. While early disclosure to healthcare practitioners is key for maternal and child health outcomes and enrolling women in early ANC, social (non) disclosure and the presence or absence of support networks also has bearings on health outcomes.

### Limitations

The limitations of this analysis are chiefly related to the targeted questions of FGDs not being focussed on concealment and with this theme arising naturally. Notably absent in FGD participants’ reasons for concealing are prohibitions on postpartum sexual relations, which are well-documented in Gambia and SSA more broadly, with mothers who become pregnant during the breastfeeding period may seek to conceal their new pregnancies while they rapidly wean the breastfeeding child [40]. A further known reason for concealment which did not appear in our FGDs is among older mothers, who fear social stigma if they are pregnant at the same time as their daughters [11, 15]. That neither motivation for concealing appeared in our FGDs further confirms the need for additional in-depth qualitative research with a larger group of participants as the absence of these reasons may have been due to the specific urban makeup of the FGD participants, that women known to conceal on these grounds were simply not captured in this round of data collection related to the design of a RCT, or the recognised shortening of postpartum abstinence timelines in recent years [41]. Finally, the overall RCT is focused on behavioural interventions, yet this present paper focuses on beliefs; without more detailed demographic data on FGD participants, particularly as relates to religion, it is difficult to approach a representative sample or offer a more fine-grained and culturally-situated analysis.

## Conclusion

Understanding the context and motivations behind concealing pregnancy during the first trimester can help direct women to early ANC. In the case of The Gambia and other countries in SSA, one part of this process of understanding should involve identifying women who perceive themselves to be vulnerable to spiritual attacks and associated miscarriages, and who therefore seek to conceal pregnancy. We suggest that providing biomedical healthcare practitioners in The Gambia with the ethnographic information to understand the local specificities of how women encounter evil spirits and these spirits’ influence on health behaviours, combined with training to identify potentially pregnant women who do not wish to disclose, can help close the early ANC access gap. Concealed pregnancy in the Gambian context may often be a considered act of care, believed to protect against acute threats to a foetus in a pregnancy’s early stage—either the threats of spirit-induced miscarriage or social ostracization associated with a pregnancy outside of marriage. Demonstrating cultural sensitivity around the rationales for concealment by recognising and respecting the actions of care demonstrated by the mother can also contribute to positive ANC experience for women; such positive experiences are identified by the WHO as an essential component of ensuring women’s continued enrolment in ANC throughout their pregnancy, even when ANC has begun late [3].

## Data Availability

The datasets generated and/or analysed during the current study are not publicly available due to reasons of sensitivity but are available from the corresponding author on reasonable request.

## References

[1] Latoff S (2020). Implementation of the new WHO antenatal care model for a positive pregnancy experience: A monitoring framework. BMJ Glob Health.

[2] Barker D, Barker M, Fleming T (2013). Developmental biology: Support mothers to secure future public health. Nature.

[3] WHO. WHO recommendations on antenatal care for a positive pregnancy experience. Geneva, World Health Organization. 2016. 28079998

[4] Haddrill R, Jones GL, Mitchell CA, Anumba DOC (2014). Understanding delayed access to antenatal care: A qualitative interview study. BMC Pregnancy Childbirth.

[5] Baer R., et al. Maternal factors influencing late entry into prenatal care: A stratified analysis by race or ethnicity and insurance status. J Maternal-Fetal Neonatal Med. 2018;3336–3342. 10.1080/14767058.2018.1463366 29631462

[6] Hill K (2007). Estimates of maternal mortalirt worldwide between 1990 and 2005: An assessment of available data. Lancet.

[7] Yaya S, Bishwajit G (2020). Predictors of institutional delivery service utilization among women of reproductive age in Gambia: A cross-sectional analysis. BMC Pregnancy Childbirth.

[8] WHO, Unicef, UNFPA, World Bank Group, and the United Nations Population Division. Trends in Maternal Mortality (2000). to 2017.

[9] Gambia Bureau of Statistics (GBoS) and ICF. The Gambia Demographic and Health Survey 2019–2020. Banjul, The Gambia and Rockville, Maryland, USA: GBoS and ICF. 2021.

[10] Stokes E, Dumbaya I, Owens S, Brabin L (2008). The right to remain silent: A qualitative study of the medical and social ramifications of pregnancy disclosure for Gambian women. BJOG.

[11] Laing (2017). Barriers to antenatal care in an urban community in the Gambia: An in-depth qualitative interview study. Afr J Reprod Health.

[12] Drammeh B, Hsieh CJ, Liu CY, Kao CH (2018). Predictors of antenatal care booking among pregnant women in The Gambia. African Journal of Midwifery and Women’s Health.

[13] Lowe M (2017). Social and cultural barriers to husband’s involvement in maternal health in rural Gambia. Pan Afr Med J.

[14] Lowe M, Chen DR, Huang SL (2016). Social and cultural factors affecting maternal health in rural Gambia: An exploratory qualitative study. PLoS ONE.

[15] Jaiteh et al. ‘Some anti-malarials are too strong for your body, they will harm you.’ Socio-cultural factors influencing pregnant women’s adherence to anti-malarial treatment in rural Gambia. Malaria J. 2016;15:195. 10.1186/s12936-016-1255-0 PMC4827243 27068760

[16] Rerimoi AJ, Niemann J, Lange I, Timaeus IM (2019). Gambian cultural beliefs, attitudes and discourse on women’s health and mortality: Interviewer’s perspectives. PLoS ONE.

[17] Vasan SK, Jobe M, Mathews J, et al. Pregnancy-related interventions in mothers at risk for gestational diabetes in Asian India and low and middleincome countries (PRIMORDIAL study): protocol for a randomised controlled trial BMJ Open. 2021;11:e042069. 10.1136/bmjopen-2020-042069. 10.1136/bmjopen-2020-042069 PMC7893661 33597136

[18] Breen RL (2006). A practical guide to focus-group research. J Geogr High Educ.

[19] Gambia Bureau of Statistics. Integrated Household Survey 2015/16, Volume II – Socio-economic Characteristics. Banjul, The Gambia. 2017.

[20] Ibid, p. 45.

[21] Saine A. Culture and customs of Gambia. Santa Barbara: Greenwood. 2012.

[22] Braun V, Clarke V (2006). Using thematic analysis in psychology. Qual Res Psychol.

[23] Sawyer A (2011). Women’s experiences of pregnancy, childbirth, and the postnatal period in The Gambia: A qualitative study. Br J Health Psychol.

[24] Dierickx et al. PloS One. 2019.

[25] Chant S, Evans A (2010). Looking for the one(s): Young love and urban poverty in the The Gambia. Environ Urban.

[26] Chapman S (2018). To make one’s name famous: Varietal innovation and intellectual property in The Gambia. Am Ethnol.

[27] Bell AJ (2020). ‘This sickness is not hospital sickness’: A qualitative study of the evil eye as a source of neonatal illness in Ghana. J Biosoc Sci.

[28] Chapman R (2011). Family Secrets: Risking Reproduction in Mozambique.

[29] Chapman R (2004). A nova vida: The commoditization of reproduction in central Mozambique. Med Anthropol.

[30] Chapman R (2006). *Chikotsa*—Secrets, silence, and hiding: Social risk and reproductive vulnerability in central Mozambique. Med Anthropol Q.

[31] Chapman R (2003). Endangering safe motherhood in Mozambique: Prenatal care as pregnancy risk. Soc Sci Med.

[32] Ibid, p. 362

[33] Hough C (2008). Re/producing mothers: Structure and agency in Gambian *kanyaleng* performances. Ethnology.

[34] Dierickx S, Rahbari L, Longman C, Jaiteh F, Coene G (2018). ‘I am always crying on the inside’: A qualitative study of the implications of infertility on women’s lives in urban Gambia. Reprod Health.

[35] O’Neill S, Clarke E, Grietens KP (2017). How to protect your new-born from neonatal death: Infant feeding and medical practices in the Gambia. Women’s Stud Int Forum.

[36] O’Neill S (2015). Foul wind, spirits and witchcraft: Illness conceptions and health-seeking behaviour for malaria in the Gambia. Malar J.

[37] Tighe SM, Lalor JG (2019). Regaining agency and autonomy: A grounded typology of concealed pregnancy. J Adv Nurs.

[38] Tighe SM, Lalor JG (2015). Concealed pregnancy: A concept analysis. J Adv Nurs.

[39] Gatrell C (2011). Policy and the pregnant body at work: Strategies of secrecy, silence and supra-performance. Gend Work Organ.

[40] Bledsoe C, Hill AG, Basu AM, Aaby P (2004). Social Norms, Natural Fertility, and the Resumption of Postpartum ‘Contact’ in The Gambia. The Methods and Uses of Anthropological Demography.

[41] African SB, Changes F, Groth H, May JF (2017). In Africa’s Population. Search of a Demographic Dividend.

